# Primary Mucosal Melanoma of the Lip With GNA11 Mutation in a 23-Year-Old Pregnant Woman

**DOI:** 10.7759/cureus.38581

**Published:** 2023-05-05

**Authors:** Shivam Khatri, Simon Kashfi, Stephanie Griffin

**Affiliations:** 1 Medical School, City University of New York (CUNY) School of Medicine, New York City, USA; 2 Internal Medicine, Donald and Barbara Zucker School of Medicine at Hofstra/Northwell, Hempstead, USA

**Keywords:** pregnant woman, cancer immunotherapy, lip, gna 11 mutation, mucosal melanoma

## Abstract

Mucosal melanoma represents a small proportion of all melanoma cases and is associated with a worse prognosis. Primary malignant melanoma of the lip (PMML) is far less common, and only a few cases have been reported since 1997, most commonly in China, Japan, Uganda, and India. Most of these cases have been associated with the gene C-KIT. As a result, treatment guidelines surrounding mucosal melanoma are unclear, especially in complicated populations such as pregnant women. Mutations in theGNAQ and GNA11 genes have been found to be associated with uveal melanoma, while they are rarely associated with mucosal melanoma. We present the case of a 23-year-old pregnant woman who was found to have likely primary malignant melanoma of the lip that metastasized to the left jaw, neck, breast, lungs, and ovaries and was found to be positive for both the BRAF-MLL3 and GNA11 mutation.

## Introduction

According to estimates, 35% of women diagnosed with melanoma are of childbearing age. As a result, current trends suggest that the incidence of pregnancy-associated melanoma is likely to increase in the future [1]. However, the effect of pregnancy on melanoma is not well understood, and as a result, the current management of melanoma during pregnancy is not well defined [1]. To complicate matters further, mucosal melanoma in pregnancy is not well documented in the literature, and there is a lack of information regarding its management.

Mucosal melanoma was first described in 1856 by Weber et al. and was classified as its own distinct disease in 1869 [2]. They are most commonly found in the mucosal membranes lining the respiratory, gastrointestinal, and genitourinary tracts and account for only 0.8%-3.7% of all melanoma cases [3]. Even more surprising is the fact that mucosal malignant melanoma arising from the head and neck region accounts for only about 0.2% of all melanomas [4]. The most common head and neck mucosal melanomas occur in the maxillary anterior gingival area; however, they are also seen in the buccal mucosa, mandibular gingiva, tongue, base or oral cavity, and lips [4,5]. These melanomas have been most commonly found in countries such as China, Japan, Uganda, and India, while being rarely reported in the United States [6]. In 2015, a review conducted by Sharma Lamichhane et al. identified fewer than 50 reported cases of primary malignant melanoma of the lip and fewer than 20 cases of mucosal melanoma of the lip after 1997 [5]. We present another case in which a 23-year-old female was found to have likely PMML that had metastasized to the left jaw/neck, breasts, lungs, and ovaries. Genetic testing was found to be positive for BRAF and GNA11 mutations. To our knowledge, B-Raf (BRAF) is found in 3% of all mucosal melanoma cases, and GNA11 has been found in about 10% of cases. 

## Case presentation

The patient was a 23-year-old woman with no past medical history who initially noticed a right neck mass one year prior to presentation. The patient reported that the neck mass had started under the left jaw and extended to the neck over the course of six months. The mass was biopsied and was positive for melanoma. In addition, the patient reported that she had a 7 cm pigmented lesion in the mid-upper gingiva extending to the palate that had been growing and darkening over the last eight years. This lesion was also biopsied and showed an increased number of single melanocytes with Melan-A staining in the basal layer. A positron emission tomography (PET) scan was performed and showed no distant malignancy. The mass was subsequently removed, and she was started on radiation therapy postoperatively. However, radiation therapy was stopped due to side effects. She was unable to eat or drink due to pain. Despite stopping radiation, she was persistently nauseous, so she tested herself for pregnancy, which turned out to be positive. A plan was initially put in place for her to start immunotherapy with nivolumab and ipilimumab; however, this was not carried out as a result of her pregnancy. Over the next month, the patient noticed a lump on her right upper chest, which prompted a visit to her primary care physician, who also noted a mass in the left chest. She was referred for an ultrasound of her breasts, which showed bilateral breast masses with a breast imaging-reporting and data system (BI-RADS) score of five. A biopsy was performed, which was found to be positive for metastatic melanoma.

**Figure 1:**
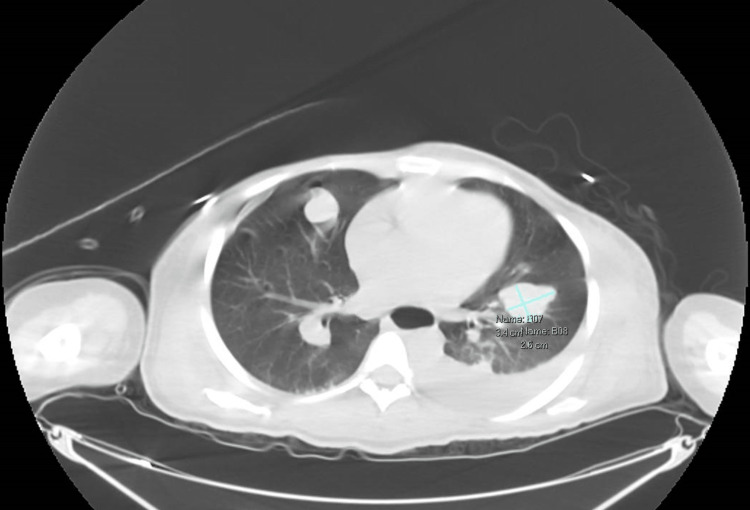
Multiple bilateral pulmonary nodules and masses compatible with metastatic disease, measuring up to 3.4 cm in the left upper lobe

She was seen by obstetrics and gynecology for an evaluation of termination of pregnancy at 15 weeks. Ultimately, she decided to prioritize the optimal gestation and future delivery of her child. After a multidisciplinary meeting to discuss the patient's care, it was decided that systemic chemotherapy with carboplatin and paclitaxel would be the most appropriate treatment until the fetus reaches about 34 weeks. Both medications were used since the benefits were deemed to outweigh the risks to the fetus. At that point, the patient would be transitioned to immunotherapy. After her first treatment, the bilateral breast lesions decreased slightly in size, with a corresponding decrease in pain. After her second treatment, her bilateral breast masses were larger in surface area but softer. The masses were heavy and caused significant impairment in quality of life, so a breast resection was performed. A magnetic resonance image (MRI) postoperatively showed bilateral pulmonary metastases increasing in size compared to a computed tomography (CT) scan the month prior (Figure 1).

**Figure 2:**
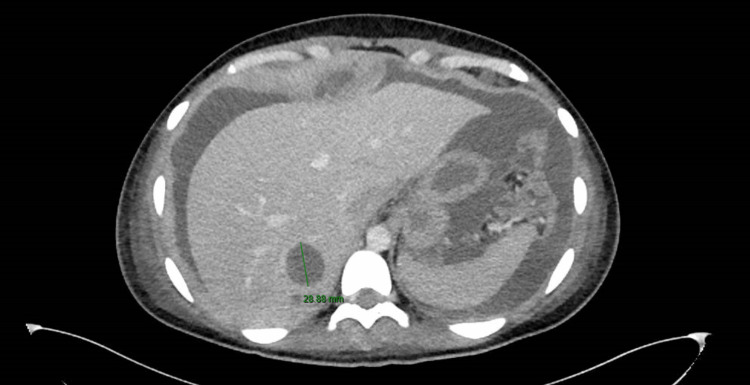
A 2.9 cm hypoattenuating lesion in the right hepatic lobe

**Figure 3:**
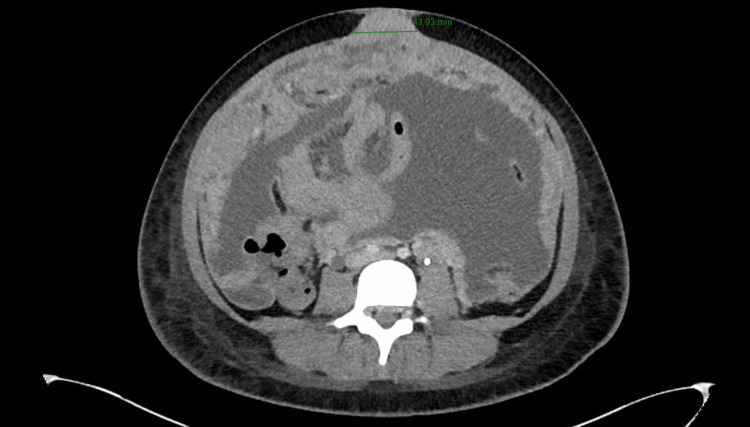
A 4.4 cm abdominal wall implant at the level of the umbilicus. Peritoneal nodularity is also seen.

The CT also showed metastatic tumor implants involving the right ovary, new peritoneal carcinomatosis in the deep pelvis (Figure 2), and hepatic metastases (Figure 3).

In addition, the patient underwent genetic testing, which confirmed that she was positive for GNA11 Q209H and BRAF-MLL3 with rearrangement in intron 9 and amplification.

Another multidisciplinary team meeting was held to re-evaluate treatment due to the rapid progression of the disease. The decision was made to initiate two cycles of immunotherapy prior to the delivery of the fetus. Unfortunately, the patient was subsequently sent to the hospital for gastrointestinal symptoms and vomiting. Imaging showed rapid progression of the melanoma, increasing the risk of delivering the fetus. Surgical delivery was performed at 28 weeks gestation, with plans to initiate immunotherapy after. She gave birth to a boy, who went to the neonatal intensive care unit for further care.

The patient presented to the emergency department on post-op day 16 with decreased oral intake and increased serous drainage from her surgical incision site. She was found to have *Escherichia coli *(*E. coli*) urosepsis and bacteremia, which were treated with ceftriaxone. A CT scan of the abdomen redemonstrated metastatic disease without post-op collections, and thus the etiology of sepsis was attributed to a urinary source. She continued to have low-grade fevers in the setting of new ascites, for which a paracentesis was performed with a total neutrophil count over 300, meeting the criteria for spontaneous bacterial peritonitis. However, body cell culture remained negative, and the elevated cell count was ultimately attributed to underlying carcinomatosis.

On hospital day 11, a rapid response was called due to a new onset of acute metabolic encephalopathy. She was intubated for airway protection and brought to the medical intensive care unit for further management. A CT of the head was negative for acute pathology, and an electroencephalogram revealed seizure activity for which levetiracetam was initiated. Despite broad-spectrum antibiotic treatment and negative blood cultures, the patient remained febrile (temperatures as high as 105.6F) with a rising lactic acidosis of unclear source but thought to be related to underlying malignancy progression. She required increasing vasopressor support and was subsequently found to be in obstructive shock secondary to abdominal compartment syndrome. Oncology was consulted and offered no disease-modifying treatment, and a family meeting was arranged to discuss the prognosis and goals of care. The transition was made to comfort care, and the patient ultimately expired on hospital day 13.

## Discussion

Mucosal melanomas are associated with a worse prognosis than cutaneous melanomas, in part because checkpoint inhibitors have been successful in treating cutaneous melanoma but have not shown the same efficacy in treating mucosal melanoma. Mucosal melanoma has also been found to be largely asymptomatic. In fact, most people do not inspect the oral mucosa unless they encounter swelling, a lack of dental mobility, ulcerations, paresthesia, or bleeding [5]. Earlier presentations of mucosal melanomas include variable-sized pigmented macules, while long-lasting lesions are found to be nodular or pedunculated and may vary in color. In about one-third of cases, mucosal melanomas may be lighter or near normal tissue color [5]. In this patient, a pigmented mouth lesion was found that had been growing and darkening over the last eight years. In fact, she only presented to the hospital when she noticed a neck mass that had started under her left jaw. Ultimately, this mass prevented her from eating or drinking without pain.

Though there have been over fifty reported cases of primary malignant melanoma of the lip, only a handful have been associated with the GNA11 mutation. In addition, the patient was found to test positive for the BRAF-MLL3 mutation. Kim et al. report a case of a 59-year-old male who was diagnosed with mucosal melanoma during hemorrhoid evaluation. A molecular analysis of the tumor was done, and it was found to be positive for a GNAQ/GNA11 mutation with a substitution of glutamine to proline in codon 209 (Q209P) [7]. In contrast, we present the case of a pregnant patient found to have mucosal melanoma of the lip who had a substitution of glutamine to histidine in codon 209 (Q209H). Though 80% of all uveal melanomas are associated with a GNA11 mutation, our patient lacked any ophthalmologic history or signs and symptoms of ocular melanoma.

The alterations in intracellular pathways caused by mutations can create targets for treatment. BRAF is an important molecule in the RAS-RAF-mitogen-associated protein kinase pathway and is associated with 50%-60% of cutaneous melanoma cases, while it is only found in about 5% of mucosal melanomas [7,8]. This patient was found to have a BRAF mutation. On the other hand, 14% of mucosal melanomas harbor a mutation in C-KIT, which is a receptor tyrosine kinase that acts as a receptor for stem cell factor ligand [7,8]. In fact, the frequency of C-KIT mutations varies by anatomical site, as mucosal melanomas of the vagina have shown a higher prevalence of such situations as opposed to those of the head and neck [8]. Our patient was found to be positive for the GNA11 mutation, which are alpha subunits of heterotrimeric G proteins that couple seven transmembrane domain receptors to intracellular signaling pathways. Mutations in the GNAQ/GNA11 are associated with the MAPK pathway, which is also activated by BRAF mutations [7]. 

When it comes to treating mucosal melanomas, most authors advocate for wide local excision, in which clear margins are achieved with or without lymph node dissection [4,9]. However, complete resection of mucosal melanomas is difficult to achieve as they are often diagnosed at advanced clinical stages, and wide negative margins may be difficult due to anatomic constraints [10]. As a result, neoadjuvant therapy for regional metastatic disease may improve clinical outcomes over surgical resection. In fact, a phase two trial published recently in the New England Journal of Medicine found that event-free survival was significantly longer among those who received pembrolizumab both before and after surgery than among those who received adjuvant pembrolizumab alone for resectable stage III or IV melanoma [11].

Anti-PD-1/anti-CTLA4 combinations such as a mix of nivolumab and ipilimumab have been used in multiple cases of mucosal melanoma, though their efficacy has been shown to be lower than in cutaneous melanomas [3,10,12]. In this case, the patient was offered this cocktail of immunotherapy; however, her pregnancy complicated matters. There have been a few case reports in which nivolumab or ipilimumab with denosumab have been shown to be effective in the treatment of mucosal melanomas [13,14]. Such immunotherapy has been classified as pregnancy category D by the Food and Drug Administration, which means that the drugs in this class may be used in pregnancy if the benefits to the mother outweigh the risk to the fetus [13,14]. As a result, immune checkpoint inhibitors are discouraged during pregnancy, as one case series showed that 88.9% of pregnant patients on immunotherapy had premature onset of labor, while 71.4% had other complications such as intrauterine growth restriction, placental insufficiency, and lower fetal heart rates [14]. Though mucosal melanoma has been associated with poor outcomes, combination anti-PD-1/anti-CTLA4 therapy has been shown to be one of the most effective strategies for treating mucosal melanoma. Combined therapies include vemurafenib for BRAF V600E mutations and imatinib for C-KIT mutations. Since our patient was not found to have a mutation in the C-KIT gene, imatinib was not used [6,7]. Since GNAQ mutations are drivers of mitogen-activated protein kinase (MAPK) activation similar to BRAF, new studies have identified selumetinib (a selective MEK inhibitor) as a possible therapy in metastatic uveal melanoma [15]. Furthermore, carboplatin and paclitaxel, two chemotherapeutic agents that were used in this case, have shown one-year overall survival and three-month progression-free survival in the nivolumab-refractory setting in one trial [16]. Reasonable responses were also seen in another trial, hence their use in our pregnant patient with PMML [17].

This case also serves as an ethical dilemma. The patient had to weigh the principles of beneficence and nonmaleficence in her treatment of both herself and her child. Her outcome may have improved with early termination of pregnancy and the initiation of immunotherapy. However, she exercised autonomy in her decision to continue with the pregnancy. This is an important dilemma that many patients, families, and oncologists face when undergoing treatment.

## Conclusions

Mucosal melanomas have a poor prognosis and lack robust literature. Physicians should identify risk factors for melanoma and perform skin exams to identify potential harmful lesions early. Mucosal melanomas are often diagnosed late, leading to poor outcomes. Anti-PD-1/anti-CTLA4 therapies are preferred as first-line therapies, though further research is warranted.
